# Effects of Caffeine on Event-Related Potentials and Neuropsychological Indices After Sleep Deprivation

**DOI:** 10.3389/fnbeh.2020.00108

**Published:** 2020-06-22

**Authors:** Xuewei Chen, Liwei Zhang, Danfeng Yang, Chao Li, Gaihong An, Jing Wang, Yongcong Shao, Rong Fan, Qiang Ma

**Affiliations:** ^1^Department of Operational Medicine, Tianjin Institute of Environmental and Operational Medicine, Tianjin, China; ^2^Key Laboratory of Behavioral Science, Institute of Psychology, Chinese Academy of Sciences (CAS), Beijing, China; ^3^School of Psychology, Beijing Sport University, Beijing, China; ^4^Central Laboratory, Xi Qing Hospital, Tianjin, China

**Keywords:** caffeine, ERP, total sleep deprivation, Go/ No-Go, reaction time (RTs)

## Abstract

**Objective**: Caffeine is a central nervous system stimulant that can effectively alleviate brain fatigue and low cognitive efficiency induced by total sleep deprivation (TSD). Recent studies have demonstrated that caffeine can improve subjective attention and objective behavioral metrics, such as arousal level, reaction time, and memory efficiency. However, only a few studies have examined the electrophysiological changes caused by the caffeine in humans following sleep disturbance. In this study, an event-related potential (ERP) technique was employed to measure the behavioral, cognitive, and electrophysiological changes produced by caffeine administration after TSD.

**Methods**: Sixteen healthy subjects within-subject design performed a visual Go/No-Go task with simultaneous electroencephalogram recording. Behavioral and ERP data were evaluated after 36 h of TSD, and the effects of ingestion of either 400 mg of caffeine or placebo were compared in a double-blind randomized design.

**Results**: Compared with placebo administration, the Go hit rates were significantly enhanced in the caffeine condition. A simple effect analysis revealed that, compared with baseline, the Go-P2 amplitude was significantly enhanced after TSD in the caffeine consumption condition. A significant main effect of the drug was found on No-Go-P2, No-Go-N2 amplitude, and Go-P2 latency before and after TSD.

**Conclusion**: Our findings indicate that caffeine administration has acute effects on improving the efficiency of individual automatic reactions and early cognitive processes. Caffeine was related to the preservation of an individual’s arousal level and accelerated response-related decisions, while subjects’ higher-level recognition had limited improvement with prolonged awareness.

## Introduction

Sleep deprivation (SD) is common in the current society, with a prevalence of approximately 35% (Bandyopadhyay and Sigua, 2019). SD refers to the state that occurs when there is a loss of sleep and increased wakefulness that is maintained for a certain time (Roca et al., 2012; Kusztor et al., 2019), and total sleep deprivation (TSD) is the elimination of sleep for some time (at least one night) to significantly prolong wakefulness (Reynolds and Banks, 2010). TSD is one of the main reasons for a low arousal level, reduced cognitive function, and increased reaction times, among other things. Since TSD has serious effects on human cognitive brain function, studies on interventions for mitigating the impact of TSD have become increasingly prevalent in this research field.

Recently, there has been a trend toward the use of caffeine (1,3,7-trimethylxanthine) to alleviate the effects of TSD and maintain arousal levels (Spaeth et al., 2014; Burrows et al., 2020). Worldwide, caffeine is the most widely consumed central nervous stimulant (Colombo and Papetti, 2020). Caffeine has been classified by pharmacologists as a central nervous system stimulant affecting, with increasing doses, the cortex, the medulla, and finally the spinal cord (Arnaud, 1987). Caffeine acts in the brain as a non-specific potent inhibitor of the actions of A1 and A2 Adenosine receptors (Ribeiro and Sebastiao, 2010; Nehlig, 2016). It seems particularly effective in improving alertness in situations of reduced arousal. Caffeine maintains a higher dopamine concentration especially in those brain areas linked with “attention.” Depending on the neurotransmitter system, caffeine can affect different brain areas with different functions (Meeusen et al., 2013). Usually, caffeine has delayed effect about 3–4 h of half-life (Knutti et al., 1981, 1982; Nehlig, 2016), caffeine’s behavioral effects and the significant increase in psychomotor performance it causes have been documented in a large body of literature, in addition to improvements in attention- (Temido-Ferreira et al., 2019; Alasmari, 2020; Franceschini et al., 2020; Irwin et al., 2020; Jahrami et al., 2020), mood-, and vigor-based tasks (Dietz and Dekker, 2017; Shabir et al., 2018; Alasmari, 2020). Moreover, Beaumont et al. (2005) found that the action of caffeine both shortened response times and reduced the number of errors on psychomotor tests, which indicates that caffeine has a global action on information processing and divided attention management (Beaumont et al., 2005; Wilhelmus et al., 2017).

Although caffeine has been studied for more than a 100 years, more research is necessary to better understand how brain activity is affected by caffeine consumption (Meng et al., 2017; van Son et al., 2018; Franco-Alvarenga et al., 2019; Tarafdar et al., 2019; Ueda and Nakao, 2019). Electrophysiological technology with event-related-potential (ERP) component detection, such as P50, N200, and P300, has been used for the measurement of brain activity. This technology allows for the measurement of neuroelectric activity related to cognitive processes, such as attention allocation and activation of short-term memory. Specific electrical patterns as measured using electroencephalography (EEG) can be evoked by sensory stimulation, such as visual and auditory stimulation. This evoked activity, or ERP, typically consists of several positive and negative peaks (Jin et al., 2015). ERPs are time-locked and can reflect both endogenously and exogenously driven cognitive processes. Concerning ERP components that reflect stimulus processing, a general arousal effect of caffeine would thus be expected to affect all components similarly, acting broadly as a stimulant amplifying all aspects of brain function (Kahathuduwa et al., 2017; Barry et al., 2019). For specific stimuli in certain response inhibition tasks, such as Go and No-Go stimuli in Go/No-Go tasks, corresponding evoked potentials can be generated during brain processing. Go-related potential changes are mainly related to automatic response processing, while No-Go-related potential changes are related to response inhibition.

Several ERP studies have examined the impact of TSD on vigilant attention during target detection and selective attention as it interacts with working and visuomotor memory (Zhang et al., 2014; Jin et al., 2015). These studies have found that TSD reduces early (~160–200 ms) or late (>250 ms) ERP component amplitudes, or delays the latencies of these components. Jin et al. (2015) found that TSD induces a dose-dependent functional decline in response inhibition (No-Go-N2 and No-Go P3 amplitudes), and 8 h of recovery sleep resulted in a partial recovery or maintenance of response inhibition (Jin et al., 2015).

Tieges et al. (2009) examined the effects of caffeine in a task-switching paradigm and reported that caffeine increased N2 amplitude, but did not affect N2 latency. By contrast, P2 and P3 latencies were reduced, with no amplitude effects, indicating the difficulty in conceptualizing such inconsistent effects between components (Tieges et al., 2009). In an auditory Go/No-Go task, Barry et al. (2007) found that a single oral dose of caffeine (250 mg) resulted in focal rather than global increases in P1, P2, and P3b amplitudes to Go stimuli with no changes in latency, suggesting that caffeine differentially improves aspects of processing related to response production and task performance (Barry et al., 2007). Within the visual Go/NoGo paradigm, ERP studies have suggested that the N2 component reflects stimulus perception (Dulinskas and Ruksenas, 2019; Song et al., 2019), cognitive control, and response inhibition (Magnuson et al., 2019; Quaglia et al., 2019). P300 is the largest positive-going peak amplitude of the waveform within a time window of 300–400 ms and is considered to represent the allocation of attentional resources to rare salient stimuli (Cote et al., 2001; Marhöfer et al., 2015). P300 amplitude and latency are thought to reflect cognitive processing, such as stimulus identification and evaluation (Feng et al., 2019; Wang et al., 2019; Gao et al., 2020; Khedr et al., 2020). Studies have also suggested that higher-order cognitive stimuli-elicited P300 components are generated from the anterior cortex, and these components reflect the response inhibition process (de Bruijn et al., 2020; Paul et al., 2020). However, Deslandes et al. (2006) and Tieges et al. (2009) have found no significant alteration of ERP indices or other neuropsychomotor results following caffeine administration after TSD, indicating that there is still a lack of knowledge of caffeine’s effects on the human brain.

By comparing ERPs related to response inhibition tasks before and after TSD, we can understand how the brain’s automatic response or response inhibition is affected by TSD. In the present study, we utilized ERP techniques to analyze behavioral, cognitive, and electrophysiological changes produced by caffeine administration after TSD. Based on previous studies, we hypothesized that TSD would induce a decrease in amplitude and a prolonged latency of the N2/P3 components. We also hypothesized that caffeine consumption would attenuate the decline in response accuracy and prolongation of reaction time (RT) caused by TSD. Because caffeine mainly enhances the alertness level of individuals, we supposed that ingesting caffeine after TSD can improve the process of automatic response and response inhibition, which will be reflected in increased amplitude and prolonged latency of ERP involving Go or No-Go stimulation. We chose 36 h of TSD to induce a moderate intensity of fatigue in subjects, to better observe the effect of caffeine intervention. To address these problems clearly, a visual Go/No-Go task with simultaneous EEG recording was used to evaluate caffeine’s effect on brain function before and after 36 h of TSD.

## Materials and Methods

### Subjects

Sixteen healthy male undergraduate students (age range 18–28 years, mean 25.9 ± 2.3 years) recruited from Beijing Normal University participated in this study. All subjects were right-handed and healthy, and we specifically excluded any potential subjects with diseases of the peripheral or central nervous system, cardiovascular disease and/or hypertension, cataracts and/or glaucoma, pulmonary problems, audiological problems, or alcohol or drug abuse. All subjects had normal vision and the standard full-length Raven’s test was employed to measure subjects’ IQ (mean 112 ± 8.7). All subjects had no psychiatric disorders (Peebles et al., 2001). All subjects scored < 60 (mean 12 ± 5) on the Symptom Checklist-90 (SCL-90; Kenemans et al., 1991). Finally, subjects were asked to be free of tobacco smoking and caffeine intake and have a regular sleep pattern with at least 8 h of sleep per night for at least 1 week before the experiment. We asked subjects about a prior history of caffeine use. Subjects who reported a prior history of caffeine intake habit (one cup per day) were excluded from our study. The experiment was fully explained to all subjects, and written informed consent was obtained before the start of the experiment. The experiment was approved by the ethics committee of the Beijing Institute of Basic Medical Science. The experiment was performed in accordance with the ethical standards of the 1964 Declaration of Helsinki. The subjects were paid $200 for participating in the study.

### Experimental Design and Task Procedures

**Figure 1:**
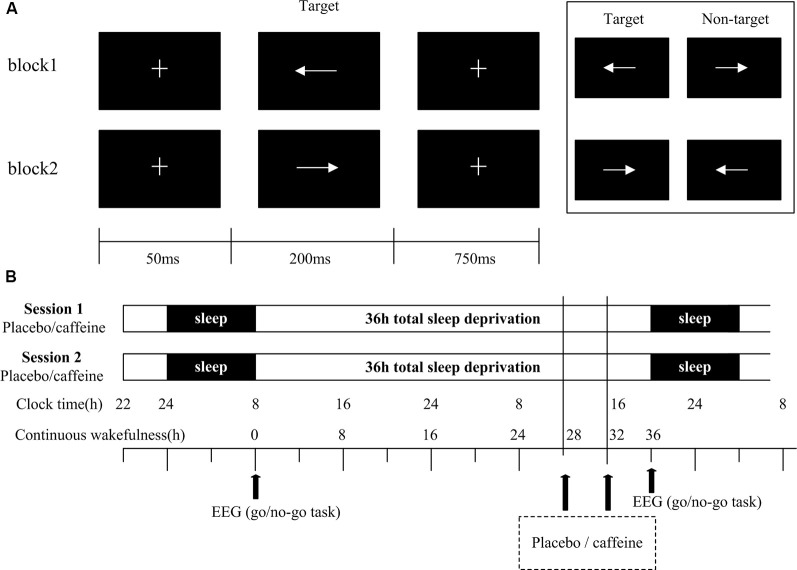
Go/No-Go task and study protocol. **(A)** Schematic representation of the Go/No-Go task, showing a single trial of two blocks. Each block consisted of 200 trials. **(B)** In the study protocol, subjects underwent two 36 h periods of total sleep deprivation (TSD). The black arrows indicate the time points of electroencephalogram (EEG) recordings and drug administration (400 mg of caffeine or placebo).

The visual Go/No-Go task was presented on a screen with a resolution of 1280 × 768 pixels, as shown in Figure 1A. At the beginning of each trial, a small white cross (+) on a black background appeared in the center of the screen for 50 ms, followed by the stimulus. Each stimulus was presented for a duration of 200 ms with an inter-stimulus interval of 750 ms. The time window for responses was <1,000 ms. The cross was displayed onscreen whenever a stimulus was not displayed. The stimulus had two arrow types (left and right, 78 × 18 pixels each, white visual stimulus on a black background) that were presented in a block task in a pseudorandom way. The task had two blocks with 200 trials in each block. In one block, the subjects were asked to respond to the left arrow [target stimulus (Go)] and withhold responding to the right arrow [non-target stimulus (No-Go)], while in the other block, the response pattern was reversed. The Go stimuli occurred with a 67% probability; the sequence of Go/No-Go stimuli is pseudorandom to ensure that No-Go stimuli do not appear in a continuous sequence. Response within 50 ms after presentation of the stimuli is regarded as invalid (Casement et al., 2006). Missed stimuli were not considered for further study. The subjects were instructed to respond as quickly as possible while maintaining a high level of accuracy and to maintain their attention on the fixation mark during the task blocks.

Subjects underwent a training session to ensure that they understood the Go/No-Go task, and to ensure that their performance was above 90%. The subjects slept for 7–9 h in a bed at the laboratory. The sleep time was assessed *via* a questionnaire and recorded by the experimenter. Subjects were tested in two sessions with a one-month interval. For the first session, the subjects arrived at the laboratory at 22:00 to ensure a full night’s sleep before TSD. At 8:00 the following morning, after a routine sleep, the subjects performed the Go/No-Go task. The subjects were not allowed to sleep for the following 36 h, during which they took either 400 mg of placebo (starch) or caffeine at the 28th hour, and the same drug was taken again at the 32nd hour. After 36 h, subjects were asked to complete the Go/No-Go task again. Subjects were accompanied and supervised by the experimenters throughout the experiment to ensure that they completed the relevant experimental tasks, such as taking medicine, testing, and maintaining wakefulness. Throughout the experiment, the subjects were required to stay in the laboratory at all times, and were only allowed to have conversations, read, play computer games, and do other non-violent activities. They were not allowed to smoke or drink coffee, hot chocolate, alcohol, or other stimulating drinks. The second session was the same as the first session, except that the subjects received the drug they did not receive in the first session. For example, if the subject received a placebo in the first session, then caffeine was taken in the second session. Subjects received either caffeine or placebo, in a randomized, double-blind design (Figure 1B). The visual Go/No-Go task was performed with simultaneous EEG recording.

### EEG Recording

The study was designed following international Pharmaco-EEG group standards. Continuous EEG recordings were obtained using a SynAmps2 amplifier (Compumedics Neuroscan, Charlotte, NC, USA). The subjects wore an Ag/AgCl electrode cap that had electrodes at the 32 sites specified by the international 10-20 system, and the reference electrodes were the digitally-linked bilateral mastoids (Duffy et al., 2013). The sampling frequency was 1,000 Hz, and the electrode impedances were maintained below 5 kΩ. The subjects were seated comfortably in a quiet, light-attenuated, and magnetic-free room. EEG was recorded from 20 monopolar derivations (Fp1, Fp2, F3, Fz, F4, F7, F8, C3, Cz, C4, T3, T4, T5, T6, P3, Pz, P4, O1, Oz, and O2).

### ERP Preprocessing

The raw EEG data were analyzed offline using Scan 4.3 (Neuroscan Products). The eye movement artifacts of the EEG data were corrected using the time-domain regression analysis method, which was implemented with Scan 4.5 software (Casement et al., 2006). Epochs with a length of 900 ms that ranged from −100 ms to 800 ms with respect to the onset of the stimuli were then extracted from the continuous EEG data. Trials with incorrect responses or RTs outside the acceptable time range (50–800 ms) were excluded. The stimuli-locked ERP was baseline-corrected for the range of −100 ms to 0 ms before stimuli onset. The range of parameters for artifact removal was from −100 ms pre-artifact to 100 ms post-artifact, and the amplitude was between −100 μV and 100 μV. A band-pass filter from 0.5 Hz to 40 Hz was then used to filter the epoch data. The frequency slope of the filter was 24 dB/oct. Stimuli-locked data averages were computed separately for each participant and each drug condition.

The ERP components P2 (120–200 ms), N2 (200–350 ms), and P3 (300–550 ms) of the stimulus trials were identified and quantified. The grand-average peak amplitudes and latencies of the three components were calculated separately at F3, Fz, F4, C3, Cz, C4, P3, Pz, and P4. These areas are the ones usually activated by the stimuli (Choo et al., 2005; Verweij et al., 2014; Jin et al., 2015; Lei et al., 2015; Feng et al., 2019; Ueda and Nakao, 2019; Wang et al., 2019; Khedr et al., 2020).

### Behavior Performance and ERP Component Analysis

The number of trials per subject for our behavioral and ERP analysis was 264 at go-trial and 136 at no-go-trial, respectively.

All behavior performance analyses were conducted using SPSS 22 software for Windows. The behavioral outcome variables included the mean RT for correct hits, hit rates (correct button presses for Go stimuli), and the percentage of false alarms (FA, incorrect button presses in response to No-Go stimuli), which were used as indices of individual behavior performance. A repeated measure ANOVA was employed to analyze the drug effects (placebo and caffeine) and the time effects (baseline and 36 h-TSD) on the behavioral data (van Son et al., 2018; Daou et al., 2019).

The repeated measure ANOVA was also used for the analysis of ERP indices. ANOVAs were performed on the P2, N2, and P3 components of the scalp electrodes in the Go/No-Go task. Greenhouse-Geisser corrections were applied when the data do not conform to the hypothesis of the spherical test.

The “eta squared” method provided by IBM SPSS 22 was employed for estimates of effect size.

## Results

### Behavioral Performance

The hit rates in Go trials showed a significant difference based on a main effect of the drug (*F*_(1,31)_ = 5.054, *p* = 0.037, ES = 0.188) and a main effect of time (*F*_(1,31)_ = 8.209, *p* = 0.009, ES = 0.273), but no interaction effects (drug × time; *F*_(1,31)_ = 1.899, *p* = 0.180, ES = 0.080). A simple effect analysis showed no significant difference in the hit rates between placebo and caffeine conditions at baseline; however, there was a significantly increased hit rate with caffeine compared with placebo after TSD (*p* = 0.028).

The RTs in Go trials showed a significant difference based on a main effect of drug (*F*_(1,31)_ = 5.541, *p* = 0.031, ES = 0.223) and a main effect of time (*F*_(1,31)_ = 5.462, *p* = 0.034, ES = 0.220). A simple effect analysis revealed a significantly increased RT in caffeine compared with that in placebo at baseline (*p* = 0.050); however, there was no significant difference after TSD.

The FA rates of No-Go trials showed no significant difference in the main effects or interaction effects (*p* > 0.05).

**Figure 2:**
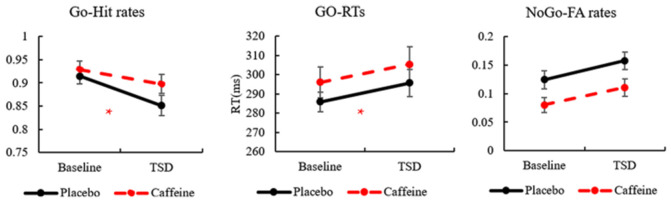
The standard errors and means of Go-hit rates, Go-reaction times (RTs), and No-Go-false alarm (FA) rates. *Caffeine vs. Placebo*, p* < 0.05.

The standard deviations and means of the Go-hit rates, Go-RTs, and FA rates before and after TSD are presented in Table 1. To more clearly observe the effects of taking caffeine, performance metrics under caffeine, and placebo conditions were compared, as shown in Figure 2.

**Table 1:** Summary of behavioral performance (mean ± deviation).

	Placebo			Caffeine		
	Baseline	36 h-TSD	*p*-value	Baseline	36 h-TSD	*p-*value
Go-hit rates	0.914 ± 0.077	0.851 ± 0.104	0.003*	0.929 ± 0.089	0.898 ± 0.098^#^	0.158
Go-RTs (ms)	285.852 ± 22.725	295.746 ± 31.467	0.006*	295.947 ± 35.466^#^	305.389 ± 40.520	0.172
No-Go-FA rates	0.124 ± 0.069	0.158 ± 0.068	0.109	0.080 ± 0.059	0.111 ± 0.069	0.085

Note: *Baseline vs. 36 h-TSD, *p* < 0.05; ^#^Placebo vs. Caffeine, *p* < 0.05.

### ERP

**Figure 3:**
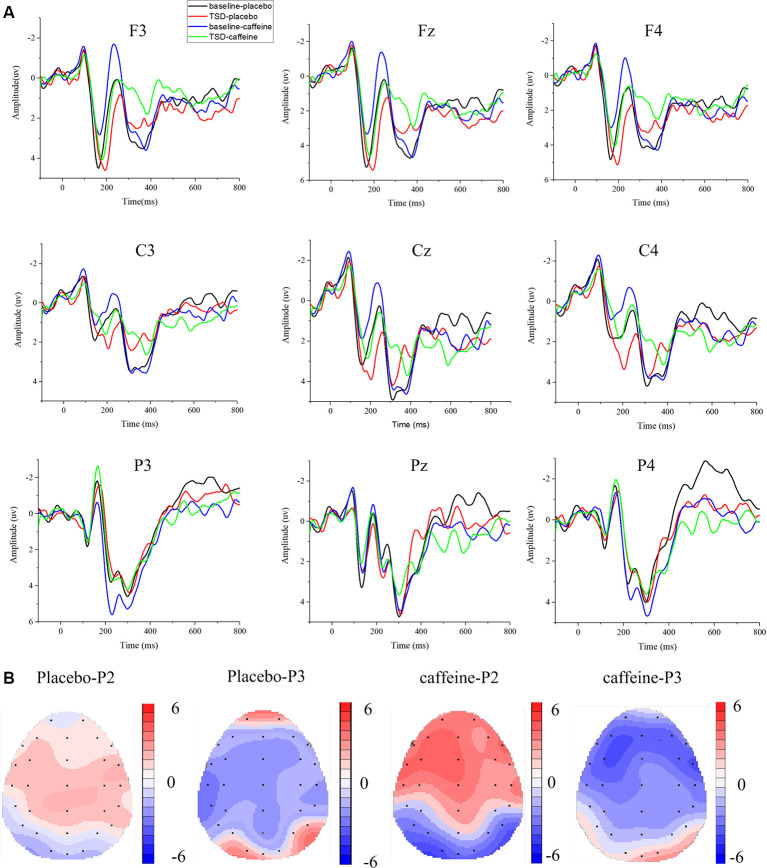
Differences between event-related potential (ERP) component amplitudes and topographic analysis following caffeine or placebo treatment at baseline and after TSD in Go trials. **(A)** Stimulus-locked average certainty ERP responses following placebo or caffeine administration at baseline and after TSD. **(B)** Significant differences in P2 and P3 components in scalp topography between baseline and after TSD. Headpoles of the paired *t*-test approach (*p* < 0.05; Bonferroni corrected) map the scalp distribution of statistical differences between baseline and after TSD (placebo-P2 most significant: Pz channel, two-tailed paired *t*-test, *t* = 2.316, *p* = 0.028; placebo-P3 most significant: C3 channel, two-tailed paired *t*-test, *t* = −3.78, *p* = 0.001; caffeine-P2 most significant: F3 channel, two-tailed paired *t*-test, *t* = 3.897, *p* = 0.01; caffeine −P3 most significant: F3 channel, two-tailed paired *t*-test, *t* = −3.082, *p* = 0.005). Colors represent the *t*-values of the statistical comparisons (color bar indicates *t*-values), and the black points represent electrodes. The larger the *t*-value, the greater the difference between the values before and after TSD. Placebo-P2: P2 changes after placebo administration; placebo-P3: P3 changes after placebo administration; caffeine-P2: P2 changes after caffeine administration; caffeine-P2: P2 changes after caffeine administration.

**Figure 4:**
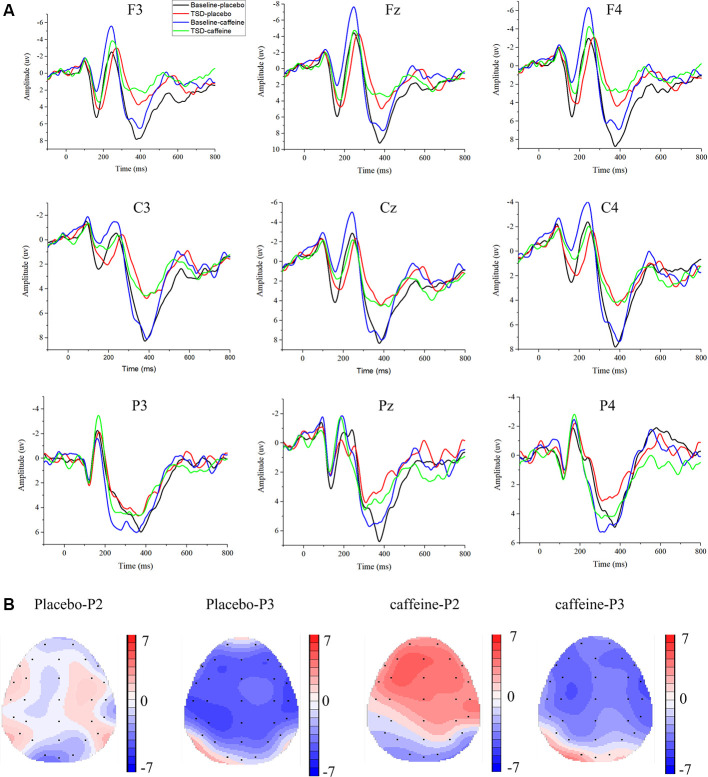
Differences between ERP component amplitudes and topographic analysis following caffeine or placebo treatment at baseline and after TSD in No-Go trials. **(A)** Stimulus-locked average certainty ERP responses following placebo or caffeine administration at baseline and after TSD. **(B)** Significant differences in P2 and P3 components in scalp topography. Headpoles of the paired *t*-test approach (*p* < 0.05; Bonferroni corrected) map the scalp distribution of statistical differences between baseline and after TSD (placebo-P2 most significant: C4 channel, two-tailed paired *t*-test, *t* = 1.987, *p* = 0.035; placebo-P3 most significant: P3 channel, two-tailed paired *t*-test, *t* = −4.056, *p* < 0.001; caffeine-P2 most significant: Fz channel, two-tailed paired *t*-test, *t* = 4.310, *p* < 0.001; caffeine-P3 most significant: C3 channel, two-tailed paired *t*-test, *t* = −4.156, *p* < 0.001). Colors represent the *t*-values of the statistical comparisons (color bar indicates *t*-values), and the black points represent electrodes. Placebo-P2: P2 changes after placebo administration; placebo-P3: P3 changes after placebo administration; caffeine-P2: P2 changes after caffeine administration; caffeine-P2: P2 changes after caffeine administration.

The means and standard deviations of the P2, N2, and P3 components’ amplitudes and latencies at the nine electrode sites in the Go trials are presented in Table 1, and the average waveforms are shown in Figure 3. The means and standard deviations of the P2, N2, and P3 components’ amplitudes and latencies elicited during the No-Go trials at the nine electrode sites are presented in Table 1, and the average waveforms are shown in Figure 4. The scalp topography shows the differences in P2/P3 before and after TSD. It can be seen from the scalp topography that the energy differences of the P2 and P3 components before and after TSD is greater after ingesting caffeine than after taking placebo. The larger changes are in frontal regions of the brain.

In Figure 3, it can be seen that, compared with the placebo condition, the P2, N2, and P3 components in the caffeine condition have larger amplitude changes before and after TSD in Go trials, especially in the frontal area (F3, Fz, and F4). To compare the effects of caffeine on the ERP components in the anterior, middle, and posterior brain regions, we performed ANOVAs of the Fz, Cz, and Pz channels located at the midline of the brain.

#### Changes in ERP Component P2

The Go-P2 amplitude in the Fz channel showed significant main effects of drug (*F*_(1,31)_ = 13.211, *p* = 0.001, ES = 0.329) and time (*F*_(1,31)_ = 6.13, *p* = 0.020, ES = 0.185); however, an interaction effect was not found (*F*_(1,31)_ = 3.719, *p* = 0.064, ES = 0.121). Furthermore, *Post hoc* multiple comparisons found that, compared with baseline, the Go-P2 amplitude was significantly enhanced after TSD in the caffeine consumption condition (*p* = 0.001). In TSD conditions, compared with placebo, caffeine caused a significant enhancement of the Go-P2 amplitude (*F*_(1,31)_ = 7.027, *p* = 0.015, ES = 0.251). Both before and after TSD, the amplitude of Go-P2 in the caffeine condition was significant smaller than that in the placebo condition (before: *p* < 0.001; after: *p* = 0.049), and the difference of amplitude between after TSD was smaller than that at baseline in caffeine condition (*p* = 0.001).

During Go trials, the Go-P2 latency in the Fz channel showed a significant difference based on the main effect of the drug (*F*_(1,31)_ = 5.360, *p* = 0.028, ES = 0.166) and a main effect of time (*F*_(1,31)_ = 25.503, *p* < 0.001, ES = 0.486); *Post hoc* multiple comparisons showed that there was no significant difference between placebo and caffeine at baseline (*p* = 0.710), however, there was a significantly shorter Go-P2 latency in the caffeine condition compared with placebo after TSD (*p* = 0.002).

ANOVAs of the Go-P2 amplitude and latency in the Cz and Pz channels did not yield significant results.

In the Fz channel, during No-Go trials, significant main effects of drug (*F*_(1,31)_ = 18.766, *p* < 0.001, ES = 0.410) and time (*F*_(1,31)_ = 8.564, *p* = 0.007, ES = 0.241) on No-Go-P2 amplitude were found; an interaction effect was also found (*F*_(1,31)_ = 8.910, *p* = 0.006, ES = 0.248). Furthermore, a simple effect analysis found that during resting wakefulness, the two baseline conditions (caffeine vs. placebo) showed significant differences in the No-Go-P2 amplitude (*p* < 0.001); there was no significant difference in the No-Go-P2 amplitude between caffeine and placebo conditions after TSD (*p* = 0.237). Moreover, compared with baseline, the No-Go-P2 amplitude was significantly enhanced in the caffeine consumption condition after TSD (*p* < 0.001).

In the Cz channel, there were significant main effects of drug (*F*_(1,31)_ = 18.004, *p* < 0.001, ES = 0.400) and time (*F*_(1,31)_ = 5.874, *p* = 0.022, ES = 0.179) on the No-Go-P2 amplitude. Furthermore, *Post hoc* multiple comparisons revealed that, compared with baseline, the No-Go-P2 amplitude was significantly enhanced in the caffeine consumption condition after TSD (*p* = 0.002). Both before and after TSD, the amplitude of No-Go-P2 in the caffeine condition was smaller than that in the placebo condition (before: *p* < 0.001; after: *p* = 0.039), and the difference of amplitude between after TSD was smaller than that at baseline in caffeine condition (*p* = 0.002).

ANOVAs of the No-Go-P2 latency in the Fz, Cz, and Pz channels and of the No-Go-P2 amplitude in the Pz channel did not yield significant results.

#### Changes in ERP Component N2

ANOVAs of the Go-N2 amplitude and latency in the Fz channel did not yield significant results.

A significant main effect of time on Go-N2 amplitude was found (*F*_(1,31)_ = 5.208, *p* = 0.031, ES = 0.162) in the Cz channel; *Post hoc* multiple comparisons found that during resting wakefulness, the two baseline conditions (caffeine vs. placebo) showed no significant difference in Go-N2 amplitude. However, there was a significant decrease in amplitude in the caffeine condition compared with placebo after TSD (*p* = 0.04).

ANOVAs of the Go-N2 latency in the Cz and Pz channels and of the Go-N2 amplitude in the Pz channel did not yield significant results.

In the Fz channel during No-Go trials, significant main effects of drug (*F*_(1,31)_ = 7.118, *p* = 0.013, ES = 0.209) and time (*F*_(1,31)_ = 10.178, *p* = 0.004, ES = 0.274) on No-Go-N2 amplitude were found along with an interaction effect (*F*_(1,31)_ = 7.062, *p* = 0.013, ES = 0.207). Furthermore, a simple effect analysis found that, compared with baseline, the No-Go-N2 amplitude was significantly enhanced in the caffeine consumption condition after TSD (*p* = 0.001). Before TSD, the amplitude of No-Go-N2 in the caffeine condition was smaller than that in the placebo condition (before: *p* < 0.001;), and the difference of amplitude between after TSD was smaller than that at baseline in caffeine condition (*p* = 0.001).

Significant main effects of drug (*F*_(1,31)_ = 13.307, *p* = 0.001, ES = 0.330) and time (*F*_(1,31)_ = 14.093, *p* = 0.001, ES = 0.343) on the No-Go-N2 latency in the Fz channel were found with no interaction effect. Furthermore, *Post hoc* multiple comparisons found that, compared with baseline, the No-Go-N2 latency shortened significantly in the caffeine consumption condition after TSD (*p* = 0.006). Both before and after TSD, the latency of No-Go-N2 in the caffeine condition was shorter than that in the placebo condition (before: *p* < 0.001; after: *p* = 0.034), and the difference of amplitude between after TSD was smaller than that at baseline in caffeine condition (*p* = 0.001).

In the Cz channel, significant main effects of drug (*F*_(1,31)_ = 3.557, *p* = 0.050, ES = 0.116) and time (*F*_(1,31)_ = 16.727, *p* < 0.001, ES = 0.383) on the No-Go-N2 amplitude were found, along with an interaction effect (*F*_(1,31)_ = 4.638, *p* = 0.040, ES = 0.147). Furthermore, a simple effect analysis found that, compared with baseline, the No-Go-N2 amplitude reduced significantly in the caffeine consumption condition after TSD (*p* < 0.001). Before TSD, the amplitude of No-Go-N2 in the caffeine condition was smaller than that in the placebo condition (before: *p* < 0.009; after: *p* = 0.924), and the difference of amplitude between after TSD was smaller than that at baseline in caffeine condition. ANOVAs of the No-Go-N2 latency in the Cz and Pz channels and of the No-Go-N2 amplitude in the Pz channel did not yield significant results.

#### Changes in ERP Component P3

There was significant main effect of time on Go-P3 amplitude in the Fz (*F*_(1,31)_ = 6.74, *p* = 0.015, ES = 0.200), Cz (*F*_(1,31)_ = 7.806, *p* = 0.009, ES = 0.224), and Pz (*F*_(1,31)_ = 8.316, *p* = 0.008, ES = 0.235) channels; however, there were no significant main effects of drug in any of the three channels. The amplitude of No-Go-P3 was the same as that of Go-P3. In the Fz channel, there was a main effect of time in the latencies of Go-P3 (*F*_(1,31)_ = 5.144*, p* = 0.032, ES = 0.160) and No-Go-P3 (*F*_(1,31)_ = 6.860*, p* = 0.014, ES = 0.203), and an ANOVA of the No-Go-N2 latency in other channels did not yield significant results.

**Figure 5:**
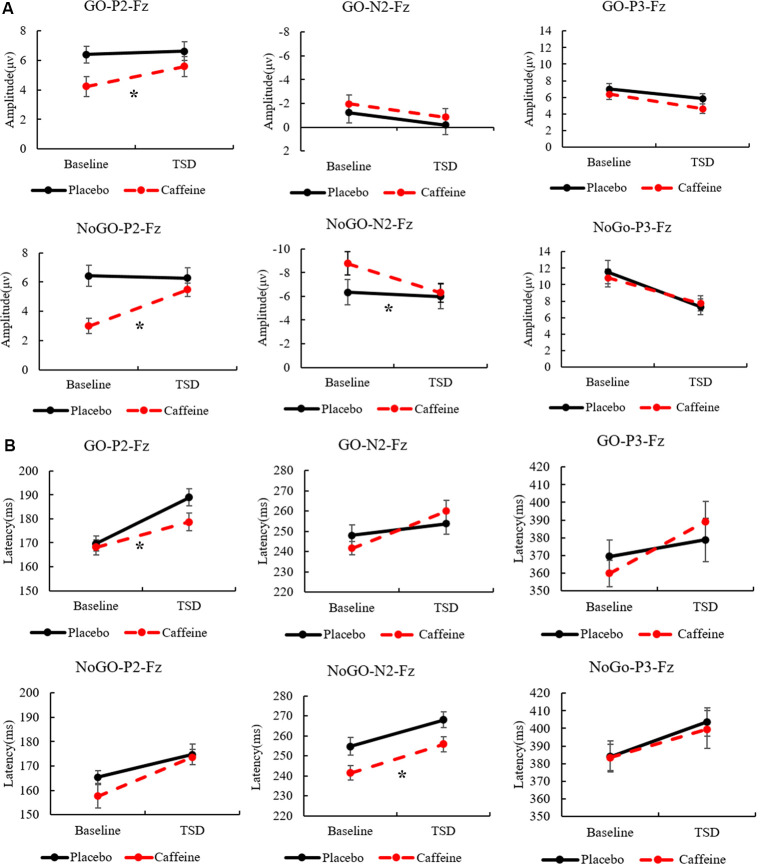
The standard errors and mean values of the amplitude **(A)** and latency **(B)** of ERP components. Solid black line, Placebo; Red dotted line, Caffeine. TSD, total sleep deprivation; Go-P2-Fz, Go trials, P2 components, Fz channel; No-Go-P2-Fz, No-Go trials, P2 components, Fz channel. *Indicates significant differences in the drug.

Combining the above results, Figure 5 shows the amplitudes and latencies of the ERP components with the main effect of the drug, highlighting the effect of caffeine on the peak ERP.

## Discussion

In the present article, we report an investigation of the effects of time (baseline and TSD) and drug (placebo and caffeine) on executive brain function using a visual Go/No-Go task with simultaneous EEG recordings. We recorded both behavioral and ERP indices in two TSD sessions to observe how automatic responses and response inhibition were altered during TSD and to what extent caffeine administration could maintain executive brain function. By examining the effects of caffeine on different ERP components, we found that the P2 ERP component in Go trials showed an increased differential wave in the TSD condition following caffeine administration compared to placebo. After TSD, the N2 and P3 components showed decreased amplitude and prolonged latency compared to baseline. However, the latency of Go-P2 in the caffeine condition was less prolonged than it was in the placebo condition; this suggests that caffeine administration may enhance cognitive processing related to response selection and inhibition.

Our study showed that the ERP components can reflect different arousal levels (TSD vs. awake). From the behavioral analysis, we found a significant decrease in hit rates and an increase in FA rates after 36 h of TSD, compared with the baseline level. Our previous study showed that the most significantly changed indices among the behavioral measurements after TSD, the RTs of Go trials, and FA rates in the No-Go trials, revealed a significant increase in performance impairment after TSD. Consistent with observations, these results revealed poor inhibitory control after 36 h of TSD and demonstrated that TSD greatly impairs higher-level cognitive functions (Tremblay et al., 2014). After caffeine administration, the hit rates in Go trials increased significantly following TSD. However, no significant changes in RTs were found in this study. These results indicate that the deterioration of performance following TSD, which was related to increased sleepiness, could be improved by caffeine administration in the Go/No-Go tasks.

An interesting finding of the present study is that the P2 component amplitude during Go trials increased after caffeine administration. Although the exact cognitive process underlying the P2 component is still widely debated, the consensus is that the P2 component reflects processes before attention. P2 is believed to reflect the post-synaptic activity of a specific neural process, and it represents aspects of higher-order perceptual processing, modulated by attention, linguistic contextual information, memory, and repetition effects (Liu et al., 2014). The exact function and neural source of the P2 component are not yet known, but some evidence has indicated that P2 may reflect general neural processes that occur when a visual (or other sensory) input is compared with an internal representation or expectation in the memory or language cortex (Stancak et al., 2018). Therefore, the larger amplitude of the P2 ERP component in Go trials may reflect the improved pre-attention brain function produced by caffeine administration after TSD.

The relevance of N2 and P3 components in individual attention processes has been established in the literature. In our study, the No-Go-P3 component showed prolonged latency after TSD, suggesting that TSD induced difficulty in inhibiting an inappropriate response. However, the increased latency of No-Go-P3 induced by TSD was not significantly improved by caffeine administration. We concluded that, rather than maintaining response inhibition, individuals maintained automatic responses. Our results regarding the No-Go-N2 and No-Go-P3 amplitudes provided evidence that the mechanism inhibiting inappropriate responses was not fully maintained, as we had speculated. These findings suggest that simple cognitive responses are easily maintained following caffeine administration, while higher-level cognitive brain functions that are related to No-Go-P3 are difficult to maintain. An alternative explanation could be that these changes are the result of an energy allocation (EA) function of sleep (Schmidt, 2014). In the EA model, the homeostatic drive to sleep is governed by an accumulation of biological deficits, or unfulfilled biological functions, favored by natural selection to utilize the state of sleep to complete such processes. Indeed, the ability to upregulate many sleep-related biological operations in waking during periods of prolonged sleep loss could explain the historical difficulty in identifying specific deficits resulting from TSD (Roca et al., 2012; Kusztor et al., 2019). During 36 h of TSD, however, the EA model predicts that energy requirements to counteract sleep deficits are directed away from advanced perception energy resources (Schmidt, 2014). The N2 and P3 ERP components measured with EEG electrodes are established neurophysiological signals with relevance to individual and working memory processes in healthy humans, patients, and even animals. In the present experiment, however, following caffeine ingestion, the P3 component measurement demonstrated some differences, and even conflicting results, from the N2 component. The variability among subjects for factors such as temperature, recent work, and the individual’s mood may account for these results. A previous study showed that the influence of caffeine on neurophysiological response is related to the individual’s alertness level (Wilhelmus et al., 2017). Findings related to ERP components deserve further exploration and investigation of the specific mechanisms responsible for these results to draw a firm conclusion in future research.

## Conclusion

These results suggest that caffeine may be beneficial to cognitive processes related to response selection and inhibition. Higher-level cognitive brain functions appeared to be improved by the administration of caffeine (Han et al., 2015; Satterfield et al., 2018). By utilizing an electrophysiological technique, the most notable results of the present study were concerning changes to the P2 component. After TSD, there was an obvious change in the N2 and P3 component amplitudes. Also, a change in the P2 amplitude was seen following caffeine ingestion. This could be explained by the fact that caffeine is related to individual arousal and accelerated response-related decisions rather than higher-level recognition (Bocca and Denise, 2006; Czisch et al., 2012). Thus, the ingestion of caffeine seems to counteract the TSD effect, which did not occur in the placebo condition. EEG studies have shown an absolute increase in the P2 amplitude after caffeine ingestion compared with the N2 and P3 components after 36 h of TSD. It reflects neuroelectric activity related to cognitive processes such as attention allocation and activation of short-term memory. Caffeine is related to the preservation of an individual’s arousal level and accelerated response-related decisions, while subjects’ higher-level recognition has limited improvement with prolonged awareness.

### Limitations

A limitation of this study was that only young male subjects were chosen. Therefore, the findings may not be generalizable to women and older people. Additionally, the sample size was small. Further, due to the one-month interval between the two TSD tests, there is a difference between the two baseline measurements, which may affect the results. The subject was under a time pressure to respond to the stimuli, which may have been faster than the actual response (Gajewski and Falkenstein, 2013). Follow-up studies should focus on the role of individual differences in ERP after TSD and caffeine consumption. The presentation time of stimuli was 200 ms, which will disturb the ERP effects because the offset potentials fail in the range of the analyzed components like the N2. Additionally, the sequential assignment of well-rested and TSD states during each testing session is a non-optimal design for studying TSD effects. Also, by the time we scanned our subjects after caffeine administration, one half-life of the caffeine had elapsed, suggesting that the drug would have been significantly eliminated from the bloodstream by the time that data were obtained. In this regard, any verdicts on the significance of the results of this study should be made with caution. Finally, individuals’ sleep-wake rhythm is a factor that may also have impacted the 36 h of TSD, and we intend to explore this in the future.

## Data Availability Statement

All datasets generated for this study are included in the article.

## Ethics Statement

The studies involving human participants were reviewed and approved by the ethics committee of the Beijing Institute of Basic Medical Science. The patients/participants provided their written informed consent to participate in this study. Written informed consent was obtained from the individual(s) for the publication of any potentially identifiable images or data included in this article.

## Author Contributions

XC designed the experiments and interpreted data. XC, LZ, and GA performed the behavior experiments. DY performed the ERP experiments. RF, CL, and JW performed processing and analysis of the data. XC and LZ wrote the manuscript. QM and YS provided the overall guidance. All authors read and approved the final manuscript.

## Conflict of Interest

The authors declare that the research was conducted in the absence of any commercial or financial relationships that could be construed as a potential conflict of interest.
